# Licensed and investigational TLR4 agonists as vaccine adjuvants: structural basis, clinical progress, and future directions

**DOI:** 10.3389/fimmu.2026.1872018

**Published:** 2026-07-16

**Authors:** Jiasheng Zhou, Bo Liu, Qiao Yang, Jingxuan Zhou, Jiahao Zheng, Yujin Chen, Lie Fu, Jianhui Du, Zhegang Zhang, Jiayou Zhang, Changgui Li

**Affiliations:** 1 Wuhan Institute of Biological Products Co. Ltd., Wuhan, China; 2 National Engineering Technology Research Center for Combined Vaccines, Wuhan, China; 3 Hubei Provincial Vaccine Technology Innovation Center, Wuhan, China; 4 National Institutes for Food and Drug Control, Beijing, China

**Keywords:** clinical translation, GLA, LPS, MPL, TLR4, vaccine adjuvants

## Abstract

The development of innovative vaccine platforms, including protein subunit and nucleic acid vaccines, has advanced rapidly and established new technical paradigms for infectious disease prophylaxis. Nonetheless, many next-generation vaccines display suboptimal intrinsic immunogenicity owing to restricted antigenic complexity, resulting in inadequate protective immunity when delivered without adjuvants. Aluminum-containing adjuvants, the most widely deployed clinical adjuvants, primarily potentiate humoral immunity but elicit modest cellular immune responses, thereby failing to satisfy the immunological demands of modern vaccine platforms. Moreover, standard vaccines often fail to confer robust protective immunity in immunocompromised individuals. Accordingly, the rational design and development of next-generation vaccine adjuvants are critical to expanding the clinical translation of innovative vaccines and enhancing immunogenicity in vulnerable populations. In recent years, multiple novel adjuvants have gained clinical approval; of these, Toll-like receptor 4 (TLR4) agonists—core immunostimulatory components of several licensed adjuvant systems—have exhibited potent immunomodulatory activity across diverse infectious disease indications. This review offers a comprehensive synthesis of contemporary TLR4-targeted adjuvants, emphasizing their evolutionary development, molecular mechanisms of action, and clinical translational landscape. We aim to furnish mechanistic insights and translational guidance for scientists optimizing current adjuvants and discovering new TLR4-based candidates.

## 1 Introduction

Vaccines constitute a cornerstone of global defense against infectious diseases. However, conventional vaccine platforms have long been constrained by intrinsic safety and immunogenicity limitations. Live-attenuated vaccines carry inherent risks of pathogen recombination and virulence reversion, representing a safety concern for immunocompromised individuals. Inactivated vaccines, while replication-incompetent, harbor heterogeneous and complex components that can trigger non-specific immune responses. Driven by rapid advances in modern biotechnology, next-generation vaccines typified by subunit and nucleic acid platforms have overcome these limitations. These platforms exhibit well-defined compositions and enable targeted delivery of key pathogen antigens. This profile eliminates the risks of viral recombination and virulence reversion linked to live-attenuated vaccines and resolves non-specific reactivity caused by complex components in inactivated vaccines, thereby establishing new avenues for enhancing vaccine safety and addressing the urgent needs of emerging infectious disease control (1).

Nevertheless, these next-generation platforms are inherently non-replicative and rely on single or narrowly defined antigenic components; many such antigens display suboptimal intrinsic immunogenicity, rendering standalone administration frequently insufficient to elicit robust, protective immune responses, creating a critical need for co-formulation with adjuvants. At present, aluminum-containing adjuvants remain the most clinically deployed class; yet they act as weak inducers of Th1-polarized cellular immunity and fail to drive robust cytotoxic T lymphocyte (CTL) responses, a core constraint that limits their efficacy against intracellular pathogens (2). Furthermore, immunocompromised individuals often fail to mount durable protective immunity following standard vaccination with alum-adjuvanted vaccines, creating a critical unmet need for novel adjuvants with enhanced immunostimulatory capacity to boost immunogenicity and compensate for innate or acquired immune defects in these high-risk populations (3, 4).

Pattern recognition receptors (PRRs) function as innate immune sentinels that detect pathogen-associated molecular patterns (PAMPs) and endogenous damage-associated molecular patterns (DAMPs), with Toll-like receptors (TLRs) representing one of the most clinically actionable PRR subfamilies (5). Unlike most other TLRs, which signal predominantly through a single adaptor—MyD88 for TLR2, TLR5, TLR7, TLR8, and TLR9, or TRIF for TLR3—TLR4 is the only family member that activates both the MyD88- and TRIF-dependent pathways (5). This dual signaling capacity enables TLR4 to potently stimulate both humoral and cellular arms of adaptive immunity. Moreover, the obligate co-receptor myeloid differentiation protein-2 (MD-2) provides a precise molecular interface for ligand engineering and signal tuning, further expanding the scope for rational adjuvant design (6). TLR4 has emerged as a particularly attractive target for the rational design of vaccine adjuvants, with monophosphoryl lipid A (MPL)—the core immunostimulatory component of clinically licensed AS01 and AS04 adjuvant systems—representing the most successful and widely deployed TLR4 agonist to date. In addition, a broad portfolio of next-generation synthetic TLR4 agonists, including glucopyranosyl lipid A (GLA) and second-generation lipid adjuvant (SLA), has exhibited favorable safety profiles and potent immunostimulatory activity in preclinical and clinical investigations, establishing them as highly promising adjuvant candidates for next-generation vaccine development.

Given the established clinical success of TLR4 agonist-based adjuvants, this review presents a historical overview of TLR4 and its agonist discovery, dissects the molecular signaling cascades triggered by ligand–receptor engagement, and systematically summarizes the global clinical development landscape of TLR4-directed adjuvants, alongside an in-depth analysis of research and translational advances of representative TLR4 agonists. The overarching goal of this review is to synthesize recent advances in this rapidly evolving field, establish a framework for the rational design and optimization of next-generation adjuvant candidates, and expedite the discovery and clinical translation of safer, more effective next-generation TLR4-targeted adjuvants.

## 2 TLR4 and Its signaling

### 2.1 TLR4

The identification of the Toll gene in *Drosophila melanogaster* by Nüsslein-Volhard and Wieschaus in 1980 laid the foundation for investigations into Toll-like receptors (TLRs) (7). In 1996, Jules A. Hoffmann and colleagues further established that the Toll signaling pathway executes an indispensable role in antifungal immune defense in *Drosophila* (5). In 1997, Charles Janeway and Ruslan Medzhitov cloned human *TLR4* and defined its core function in linking innate and adaptive immune responses (8). Structurally, TLR4 is a type I transmembrane glycoprotein comprising three evolutionarily conserved functional modules: an extracellular ligand−binding ectodomain, a hydrophobic transmembrane helix, and an intracellular Toll/interleukin−1 receptor (TIR) signaling domain. The extracellular ectodomain contains 22 tandem leucine−rich repeats that fold into a canonical horseshoe conformation, facilitating specific recognition of diverse ligands, including lipopolysaccharide (LPS); the transmembrane domain acts as a structural anchor that secures the receptor to the plasma membrane and mediates ligand−induced dimerization; the intracellular TIR domain shares high sequence homology with the interleukin−1 receptor cytoplasmic region and initiates downstream signaling cascades (9). TLR4 is constitutively expressed across a wide range of innate and adaptive immune cells, including dendritic cells, macrophages, neutrophils, natural killer cells, B lymphocytes, and T lymphocytes (10).

### 2.2 TLR4 signal transduction

In addition to its canonical ligand LPS, TLR4 detects a diverse array of pathogen−associated molecular patterns (PAMPs), including bacterial proteins and plant−derived molecules, as well as damage−associated molecular patterns (DAMPs) such as heat shock proteins released from stressed or apoptotic host cells (11–14). Mechanistically, upon entering systemic circulation, LPS forms a high−affinity complex with LPS−binding protein (LBP), which promotes the delivery of LPS monomers to the surface of monocytes and macrophages (15). The glycosylphosphatidylinositol−anchored co−receptor CD14 then captures LPS and presents it to the preassembled TLR4–MD−2 complex at the plasma membrane. Notably, while the TIR domain is highly conserved across mammals, the extracellular ligand-recognition machinery displays pronounced species divergence. LPS does not contact TLR4 directly; instead, it is fully accommodated within the hydrophobic pocket of MD-2, and the resulting MD-2/LPS complex drives TLR4 dimerization (6). Owing to species-specific residues in the MD-2 binding pocket, ligands such as lipid IVa function as agonists on mouse TLR4/MD-2 but as antagonists on the human complex (16). These interspecies differences caution against relying solely on murine models to predict adjuvant efficacy in humans and underscore the importance of validating TLR4 agonist activity in non-human primate studies or, where feasible, in clinical trials.

**Figure 1:**
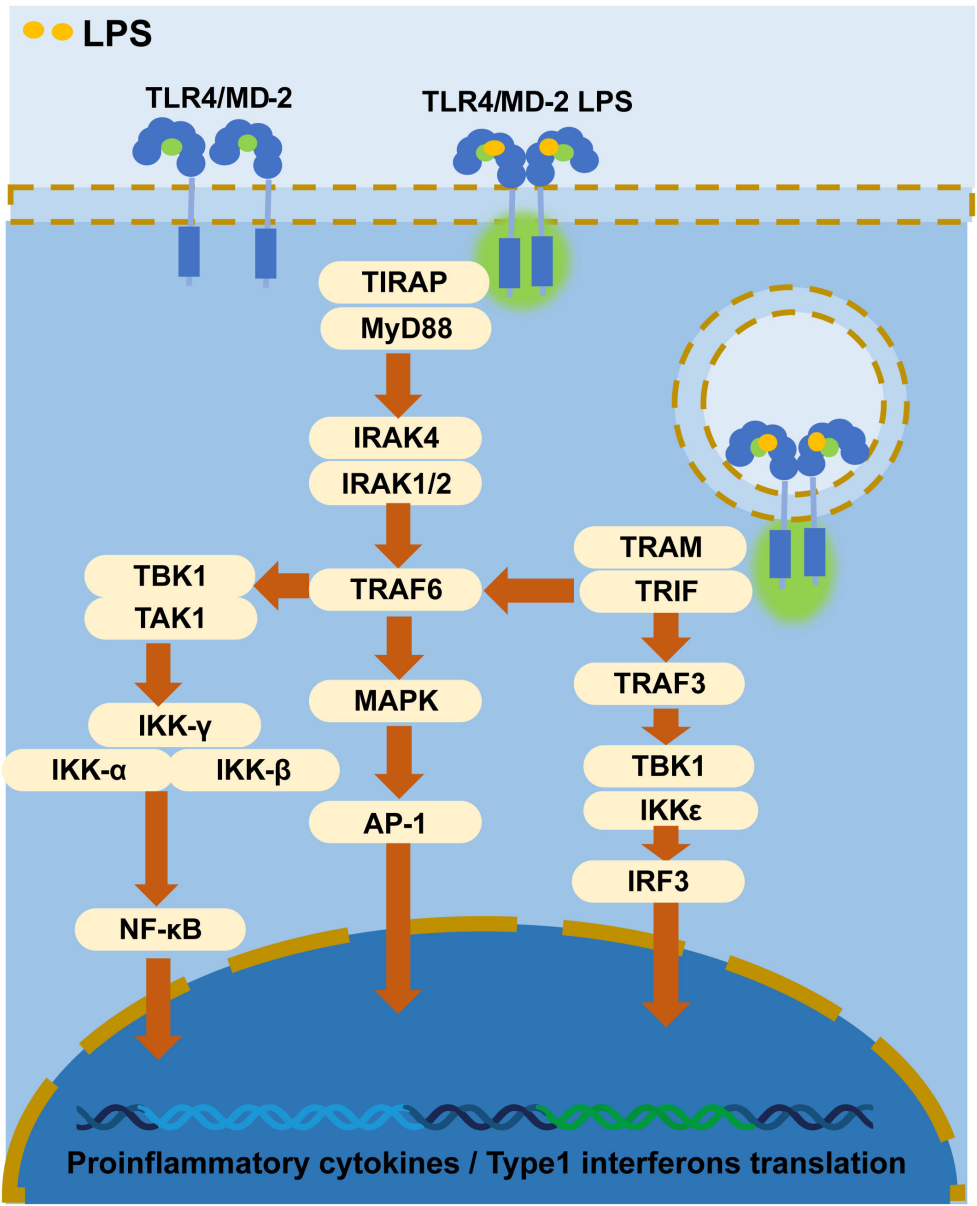
MyD88-dependent and TRIF-dependent signaling pathways activated by LPS binding to TLR4. Upon binding to the cell surface TLR4/MD-2 complex, LPS initiates two distinct signaling cascades. The MyD88-dependent pathway activates NF-κB and MAPK to induce proinflammatory cytokine production, while the TRIF-dependent pathway activates IRF3 to drive type I interferon expression. Schematic diagram of the two TLR4 signaling pathways: the MyD88-dependent pathway that activates NF-κB and MAPK to produce proinflammatory cytokines, and the TRIF-dependent pathway that activates IRF3 to produce type I interferons.

Upon activation, TLR4 triggers two discrete downstream signaling axes: the myeloid differentiation primary response protein 88 (MyD88)−dependent pathway, which recruits TIR domain−containing adaptor protein (TIRAP/Mal) to activate nuclear factor−κB (NF−κB) and mitogen−activated protein kinase (MAPK) cascades, driving proinflammatory cytokine production; and the TIR domain−containing adaptor−inducing interferon−β (TRIF)−dependent pathway, which engages TRIF−related adaptor molecule (TRAM) to activate interferon regulatory factor 3 (IRF3), promoting type I interferon and interferon−stimulated gene expression (**Figure 1**).

The MyD88−dependent cascade is critically governed by the adaptor protein MyD88. Following TLR4 activation, TIRAP/Mal is recruited to the intracellular TIR domain of TLR4 and interacts with MyD88 via homotypic TIR–TIR binding, thereby assembling the functional TLR4–MyD88 signaling complex (17–19). This active complex then recruits interleukin−1 receptor−associated kinase 4 (IRAK4)—the apical kinase in this pathway—and stimulates its phosphorylation, which further drives phosphorylation of IRAK1 and IRAK2 (20). Phosphorylated IRAK1 dissociates from the receptor complex and associates with tumor necrosis factor receptor−associated factor 6 (TRAF6), triggering its oligomerization and E3 ubiquitin ligase activity. Activated TRAF6 subsequently activates transforming growth factor−β−activated kinase 1 (TAK1) in complex with its regulatory subunits TAK1−binding protein 1 (TAB1) and TAB2. TAK1 acts as the central upstream kinase that phosphorylates and activates two discrete downstream effector modules: the inhibitor of κB kinase (IKK) complex and the MAPK family. IKK complex activation promotes IκBα phosphorylation and proteasomal degradation, enabling NF−κB nuclear translocation; meanwhile, activated MAPKs phosphorylate and activate activator protein 1 (AP−1) and additional transcription factors. Collectively, these nuclear events elicit robust transcriptional upregulation of genes encoding proinflammatory cytokines, chemokines, and costimulatory molecules (**Figure 1**).

The TRIF−dependent pathway is primarily governed by the adaptor protein TRIF (TIR domain−containing adaptor−inducing interferon−β). Upon activation, TRIF recruits tumor necrosis factor receptor−associated factor 3 (TRAF3), which then interacts with TANK−binding kinase 1 (TBK1) and IκB kinase ϵ (IKKϵ). Interferon regulatory factor 3 (IRF3) is subsequently phosphorylated by TBK1 and IKKϵ. Upon phosphorylation, IRF3 homodimerizes, translocates to the nucleus, and drives transcriptional induction of type I interferons (**Figure 1**).

Collectively, MyD88−driven proinflammatory gene transcription and TRIF−dependent type I interferon induction synergistically stimulate the production of key proinflammatory cytokines and chemokines, including tumor necrosis factor−α (TNF−α), interleukin−6 (IL−6), and interleukin−1β (IL−1β) (9, 21). These soluble mediators exert robust chemotactic activity at the vaccine injection site and draining lymph nodes, recruiting circulating innate immune cells—including monocytes, neutrophils, and immature dendritic cells—to the local inflammatory milieu and promoting their phenotypic and functional maturation. Mature antigen−presenting cells (APCs), primarily dendritic cells, efficiently interna) andnd process vaccine antigens, upregulate surface expression of major histocompatibility complex (MHC) class I and II molecules and key costimulatory ligands (CD80, CD86, CD40), and secrete additional immunomodulatory cytokines. This coordinated innate immune activation enables APCs to effectively prime naïve antigen−specific CD4+ helper T cells and CD8+ cytotoxic T lymphocytes, and to drive the differentiation of antigen−specific B cells into high−affinity antibody−secreting plasma cells and long−lived memory B cells, ultimately eliciting robust, durable, and balanced cellular and humoral protective immunity (22).

### 2.3 TLR4-mediated enhancement of vaccine-induced adaptive immunity

TLR4 activation directly compensates for a fundamental weakness of recombinant protein and subunit vaccines—their inability to deliver the innate signals needed for full DC activation and Th1 polarization—through three complementary mechanisms.

First, TLR4 activation on DCs potently upregulates co-stimulatory molecules (CD80, CD86, CD40) and, via the TRIF pathway, promotes cross-presentation of exogenous antigens on MHC class I molecules, a process essential for priming CD8^+^ cytotoxic T cells (5). Second, the dual MyD88–TRIF signaling axis drives Th1 polarization through coordinated cytokine induction: the MyD88-dependent pathway is essential for the production of interleukin-12 (IL-12), a key Th1-polarizing cytokine (23), while the TRIF-dependent pathway mediates type I interferon expression, which in turn promotes cross-presentation (21). Notably, although excessive MyD88 signaling underlies the systemic reactogenicity associated with LPS, a moderate level of MyD88 activity remains essential for the adjuvant effects of TLR4 agonists, providing the IL-12 and additional Th1-polarizing mediators necessary for efficient T cell priming. Thirdly, Stimulation of antigen-specific antibody production by B cells. TLR4 signaling can also directly act on antigen-specific B cells. Mechanistically, B-cell-intrinsic MyD88 signaling is essential for germinal center responses and early IgG production (24).In malaria vaccine studies, GLA adjuvant combined with a protective Plasmodium falciparum cysteine-rich antigen induced higher antibody titers, immunofluorescence seroconversion rates, and *in vitro* parasite growth inhibitory activity compared to Nanoalum adjuvant in mice; in rabbits, the GLA-SE formulation induced the highest titers of parasite growth-inhibitory antibodies (25). Furthermore, when combined with other types of agonists, TLR4 can exert even more powerful immunostimulatory functions; nanoparticles containing MPL and the TLR7 receptor agonist R837 increased germinal center B cell numbers by more than 5-fold in mice and significantly enhanced antibody affinity (26). Together with enhanced DC maturation and Th1 polarization, these effects enable TLR4 agonists to elicit the potent cellular and humoral immunity that recombinant protein vaccines fail to induce on their own.

## 3 TLR4 agonists

**Figure 2:**
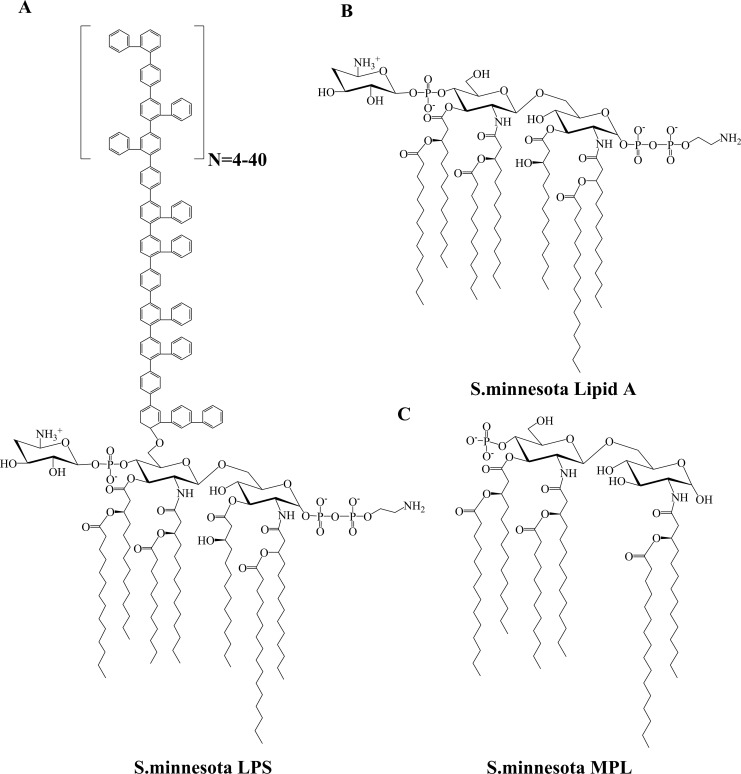
Representative structures of *Salmonella minnesota* R595 LPS, lipid A, and MPL. **(A)** LPS consists of three domains: lipid A anchor, core oligosaccharide, and O-antigen polysaccharide. **(B)** Native lipid A contains a β-1,6-glucosamine disaccharide backbone with two phosphate groups and six saturated acyl chains. **(C)** MPL is derived from LPS by controlled acid hydrolysis, which removes the 1-phosphate group and 3-O-linked acyl chain. Diagram showing the three-domain structure of *Salmonella minnesota* LPS, the structure of native lipid A with two phosphate groups and six acyl chains, and the modified structure of MPL with one phosphate group and five acyl chains.

Lipopolysaccharide (LPS), the canonical natural ligand for TLR4, is a defining structural component of the outer membrane of Gram-negative bacteria. Structurally, LPS is an amphipathic macromolecule comprising three covalently linked domains: a hydrophobic lipid A moiety, a hydrophilic core oligosaccharide, and a hypervariable O-antigen polysaccharide (**Figure 2A**) (27–29). Substantial structural heterogeneity of LPS exists across bacterial species and strains, with each domain displaying distinct degrees of evolutionary conservation. The O-antigen, the outermost and most solvent-exposed region of LPS, exhibits the highest structural diversity, differing markedly in monosaccharide composition, glycosidic linkage patterns, and polymer chain length across species and even among strains of the same species. The core oligosaccharide exhibits moderate structural variability. Lipid A, the membrane-anchoring domain of LPS, is the most evolutionarily conserved region and constitutes the minimal structural motif required for specific binding to the TLR4–MD-2 complex and downstream receptor activation (**Figure 2B**). While lipid A structure is largely conserved within a given bacterial species, it undergoes dynamic modifications in response to environmental cues, including temperature, pH, and nutrient availability (27, 30).

LPS activates most innate immune cells at picomolar to nanomolar concentrations (31, 32). However, native LPS also exhibits potent endotoxicity: even low doses can elicit local inflammation, pyrexia, and systemic inflammatory response syndrome (33). Accordingly, the direct clinical use of unmodified LPS as a vaccine adjuvant has been widely discontinued, and intensive research has focused on the development of safer alternatives that preserve potent TLR4-mediated immunostimulatory activity. Two primary strategies have been employed to address this goal: first, rational structural modification of LPS to selectively attenuate endotoxicity while preserving adjuvant activity; second, high-throughput screening to identify novel low-toxicity TLR4 agonists with distinct chemical scaffolds. At present, TLR4 agonists under adjuvant development fall broadly into two structural classes: glycolipids and non-glycolipids. Glycolipids share substantial structural homology with LPS lipid A and can be generated either by targeted chemical modification of native bacterial LPS or by *de novo* chemical synthesis. In contrast, non-glycolipids are structurally distinct from LPS, typically lacking both carbohydrate moieties and long-chain acyl groups.

### 3.1 Monophosphoryl lipid A

Since the 1970s, to harness the potent immunostimulatory properties of LPS while mitigating intrinsic endotoxicity, intensive efforts have focused on the structural modification of LPS to reduce toxicity. Ribi and colleagues developed a pioneering hydrolytic process using *Salmonella minnesota* R595 as the starting material: fermented bacterial cells underwent sequential processing steps including precipitation, washing, acid hydrolysis, and alkaline hydrolysis, yielding a heterogeneous mixture of lipid A derivatives bearing four, five, or six acyl chains and a single phosphate group, designated 3-O-deacylated monophosphoryl lipid A (3D-MPL) (**Figure 2C**) (34). Notably, 3D-MPL is the predominant clinically used isoform of monophosphoryl lipid A and is widely abbreviated simply as MPL in the vaccine adjuvant field.

MPL activates most innate immune cells at extremely low concentrations. Co-administration of MPL with antigens in aged mice elicits antigen-specific antibody titers comparable to those in young adult mice (35, 36). Notably, the endotoxicity of MPL is only 0.08% that of native LPS. Mechanistically, murine studies have shown that this attenuated toxicity is closely linked to preferential activation of the TRIF-dependent signaling pathway by MPL. In contrast to native LPS, MPL-induced immune responses are not accompanied by caspase-1 activation and downstream IL-1β secretion but are instead linked to the production of anti-inflammatory cytokines such as interleukin-10 (IL-10), which prevents excessive systemic inflammatory responses. This unique signaling profile allows MPL to preserve potent immunostimulatory activity while displaying markedly attenuated toxicity (37–39).

Despite its favorable immunostimulatory and safety profile, MPL displays very low aqueous solubility. Accordingly, tailored formulation strategies are needed to maximize its *in vivo* immunostimulatory efficacy while minimizing the risk of adverse reactogenicity. After acquiring the patent for hydrolytic MPL production, GlaxoSmithKline (GSK) launched an extensive formulation development program that successfully generated a broad portfolio of MPL-containing delivery systems, including liposomes, oil-in-water emulsions, and aluminum salt-adsorbed formulations. These efforts led to the development of a series of proprietary MPL-based adjuvant systems: AS01, AS02, AS04, and AS15. AS01 and AS04 have gained global regulatory approval for use in licensed human vaccines.

#### 3.1.1 Adjuvant system 01

The AS01 adjuvant system is a liposomal formulation containing two distinct immunostimulatory components: MPL and QS-21. It is available in two dosage forms: AS01B (50 μg MPL + 50 μg QS-21 per dose) and AS01E (25 μg of each immunostimulant per dose). QS-21 is a triterpenoid glycoside purified from *Quillaja saponaria* bark; it displays dose-dependent hemolytic activity against erythrocytes, requiring co-formulation with cholesterol and incorporation into liposomes to abrogate this adverse effect. Liposomes are vesicular structures formed by phospholipid bilayer self-assembly; hydrophobic bilayer regions encapsulate lipophilic components such as MPL, while the aqueous core accommodates hydrophilic molecules. Likewise, in the AS01 liposomal formulation, MPL is encapsulated within the liposomal lipid bilayer. This sequestration limits its direct interaction with lipopolysaccharide-binding protein (LBP) and soluble CD14 circulating in blood, further mitigating the risk of non-specific systemic activation of monocytes and macrophages (40). Mechanistically, QS-21 activates caspase-1 in subcapsular sinus macrophages, triggering inflammasome assembly and downstream maturation and secretion of proinflammatory cytokines (41). Notably, studies in non-human primates have shown that neither MPL nor QS-21 alone induces an early IFN-γ response or activates lymph node-resident NK cells and innate-like CD8^+^ T cells; these innate events require the synergistic delivery of both components within AS01 (42). This innate synergy is critical for generating the high-frequency polyfunctional CD4^+^ T cell responses that underpin the clinical efficacy of AS01-adjuvanted vaccines. Recent studies have shown that AS01 administration induces epigenetic modifications in monocyte and dendritic cell subsets, altering chromatin accessibility of key immune transcription factors, including AP-1, GATA-binding proteins, C/EBP family members, and interferon regulatory factors (43).

The AS01 adjuvant system has been included in multiple licensed human vaccines, most notably the malaria vaccine Mosquirix, the herpes zoster vaccine Shingrix, and the respiratory syncytial virus (RSV) vaccine Arexvy (**Table 1**). Mosquirix contains two antigenic components: the RTS,S antigen (a fusion protein of *Plasmodium falciparum* circumsporozoite protein fragment and hepatitis B surface antigen [HBsAg]) and supplemental recombinant HBsAg. Clinical trial results showed that a three-dose primary schedule of Mosquirix conferred 28.3% efficacy against malaria in children aged 5–17 months relative to placebo; a fourth booster dose increased efficacy to 36.3% (44). Shingrix uses a truncated varicella-zoster virus glycoprotein E (gE) as its active antigen. In clinical trials, Shingrix showed 97.2% protective efficacy against herpes zoster in adults aged ≥50 years and 91.3% efficacy in those aged ≥70 years (45, 46). Arexvy employs the prefusion conformation of RSV fusion glycoprotein (RSVPreF3) as its active antigen. In clinical studies, Arexvy significantly reduced the risk of RSV-associated lower respiratory tract disease by 82.6% in participants aged ≥60 years (47, 48). Beyond these licensed products, several AS01-adjuvanted vaccines are currently in clinical development (49, 50). Notably, the M72/AS01 vaccine induces robust immune responses in adults with Mycobacterium tuberculosis infections (**Table 2**; 50).

Of note, the AS01 adjuvant system is associated with a higher frequency and severity of local and systemic adverse events (AEs) relative to aluminum-containing adjuvants. For example, in Arexvy clinical trials, the AS01-adjuvanted group showed significantly higher adverse reaction rates than the placebo group: injection-site events included pain (60.9% vs 9.3%) and erythema (7.5% vs 0.8%); systemic events included fatigue (33.6% vs 16.1%), myalgia (28.9% vs 8.2%), headache (27.2% vs 12.6%), arthralgia (18.1% vs 6.4%), and pyrexia (2.0% vs 0.3%) (47, 48).

#### 3.1.2 Adjuvant system 04

The AS04 adjuvant system is formulated by adsorbing MPL onto aluminum phosphate. Aluminum-containing adjuvants enhance humoral immunity and boost antigen-specific antibody titers through multiple mechanisms, including antigen depot formation (sustained release) and direct immunostimulatory effects on innate immune cells. In murine models, AS04 elicited antigen-specific IgG titers 4–8 times higher than those achieved with MPL alone, while restricting proinflammatory cytokine production to the injection site and draining lymph nodes (51). Clinical data show that relative to aluminum alone, MPL adsorption onto aluminum phosphate induces rapid local cytokine expression, promotes robust APC activation, and elicits significantly higher and more durable antigen-specific antibody responses (3, 52).

The AS04 adjuvant system has been included in two licensed human vaccines: the hepatitis B vaccine Fendrix and the bivalent human papillomavirus (HPV) vaccine Cervarix (**Table 1**). Fendrix is indicated for hepatitis B virus (HBV) immunization in patients with renal insufficiency. Clinical results showed that 36 months post-vaccination, 80.4% of renal insufficiency patients receiving Fendrix achieved anti-HBs titers ≥10 mIU/ml, versus only 51.3% of those receiving a standard hepatitis B vaccine (3, 4). Cervarix is indicated for the prevention of diseases caused by high-risk HPV types 16 and 18. Cervarix provides cross-protective immunity against the five most common oncogenic HPV types (16, 18, 31, 33, 45, 52). Furthermore, a Phase III clinical trial showed that in HPV-naive individuals, Cervarix conferred 92.9% protective efficacy against HPV 16/18-related cervical intraepithelial neoplasia grade 2 or worse and adenocarcinoma *in situ* (53). In addition, clinical trial results for a herpes simplex virus type 2 (HSV-2) vaccine indicate that AS04 enhances vaccine protective efficacy (54). Notably, Phase II clinical data for the Epstein-Barr virus (EBV) gp350/AS04 vaccine showed a favorable safety profile and 78% protective efficacy against EBV-induced infectious mononucleosis (55).

**Table 1:** Licensed vaccine adjuvants containing TLR4 agonists.

Adjuvant name	Adjuvant components	Formulation type	Vaccine name	Antigen component	Target population	Key clinical data	References
AS01	MPL + QS-21	Liposome	Shingrix (Recombinant herpes zoster vaccine, CHO cell)	Varicella-zoster virus glycoprotein E (gE)	Adults aged ≥50 years (including immunocompromised individuals)	A two-dose schedule provides 97.2% protective efficacy against herpes zoster in adults aged 50 years and above.	(45, 46)
			Mosquirix (RTS,S)	*Plasmodium falciparum* circumsporozoite protein–HBsAg fusion protein (RTS,S)	Children aged 5–17 months in malaria-endemic areas	Three-dose primary vaccination yields 28.3% protective efficacy; a fourth booster dose increases efficacy to 36.3%.	(44)
			Arexvy (Recombinant RSV vaccine)	Respiratory syncytial virus prefusion F protein (RSVPreF3)	Adults aged ≥60 years	The vaccine reduces the risk of RSV-associated lower respiratory tract disease by 82.6% in adults aged 60 years and older.	(47, 48)
AS04	MPL + Aluminum salt	Alum-adsorbed	Fendrix (Adjuvanted hepatitis B vaccine)	Recombinant hepatitis B surface antigen (HBsAg)	Patients with renal insufficiency	At week 36 after vaccination, 80.4% of renal insufficiency patients achieved anti-HBs titers ≥10 mIU/ml, versus 51.3% for the conventional commercial hepatitis B vaccine.	(3, 4)
			Cervarix (Bivalent HPV 16/18 vaccine)	HPV16/18 L1 virus-like particles	Females and adolescents	In HPV-naive individuals, Cervarix exhibits 92.9% efficacy against HPV 16/18-related CIN2+ and adenocarcinoma in situ.	(53)

TLR4, Toll-like receptor 4; MPL, 3-O-deacylated monophosphoryl lipid A; QS-21, Quillaja saponaria fraction 21; CHO, Chinese hamster ovary; RSV, respiratory syncytial virus; HPV, human papillomavirus; CIN2+, cervical intraepithelial neoplasia grade 2 or worse.

Table summarizing five licensed vaccines containing TLR4 agonist-based adjuvants AS01 and AS04, including their antigen components, target populations and key clinical efficacy data.

#### 3.1.3 Adjuvant systems 02

The AS02 adjuvant system is an oil-in-water emulsion containing two immunostimulatory components: MPL and QS-21. An oil-in-water emulsion is a colloidal dispersion in which fine oil droplets are uniformly dispersed within a continuous aqueous phase. The adjuvant effects of AS02 are mediated by dual mechanisms: sustained antigen depot formation with prolonged release at the injection site, and modulation of the local immune microenvironment. Together, these mechanisms drive robust activation of innate and adaptive immunity. In a Phase II clinical trial of the RTS,S malaria vaccine in infants aged 8, 12, and 16 weeks, RTS,S/AS02 conferred 50.7% and 26.7% protective efficacy against multiple clinical malaria episodes at 12 and 18 months post-vaccination, respectively (56). In addition, AS02 has been included in several investigational HPV and HIV vaccines at various clinical stages (57, 58).

#### 3.1.4 Adjuvant systems 15

The AS15 adjuvant system is a liposome-based formulation containing three immunostimulatory components: MPL, QS-21, and CpG oligodeoxynucleotides (CpG ODN). CpG ODN are synthetic oligodeoxynucleotides containing unmethylated CpG dinucleotide motifs. Like MPL, CpG ODN are recognized as PAMPs and bind Toll-like receptor 9 (TLR9); this interaction triggers inflammatory signaling, promotes antigen-specific antibody and CD8^+^ cytotoxic T lymphocyte generation, and amplifies adaptive immunity (59). The AS15 adjuvant system has been included in multiple cancer vaccine candidates. A recombinant melanoma-associated antigen 3 (MAGEA3) vaccine was developed for the treatment of melanoma and other malignancies (60–62). However, two Phase III clinical trials showed that the AS15-adjuvanted MAGEA3 vaccine, used as adjuvant therapy after surgical resection, did not improve overall survival in patients with melanoma or nonsmall cell lung cancer (**Table 2**; 62, 63).

**Table 2:** Clinical development progress of adjuvants containing TLR4 agonists.

TLR4 agonist	Adjuvant name	Additional adjuvant components	Formulation type	Target pathogen/disease in clinical trials	Clinical trial phase	Participant characteristics	Vaccine active component/adjuvant	Key clinical data	Publication year	References
MPL	AS01	QS-21	Liposomal formulation	Mycobacterium tuberculosis	Phase II	HIV-positive individuals aged 16–35 years with well-controlled HIV infection in South Africa	Mtb32A+Mtb39A/AS01 E-4	The vaccine demonstrated a favorable safety profile and robust immunogenicity.	2025	(50)
				HIV	Phase I	Healthy adults	BG505 SOSIP.v4.1-GT1.1/AS01	The vaccine induced VRC01-class broadly neutralizing antibody precursors at high frequencies in the majority of vaccine recipients.	2025	(49)
	AS04	Aluminum hydroxide	Aluminum salt-adsorbed preparation	Herpes Simplex Virus Type 2 (HSV-2)	Phase II	Healthy females aged 10–17 years	gD2/AS04	The vaccine exhibited favorable safety and tolerability profiles and robust immunogenicity in girls aged 10–17 years, regardless of age or pre-vaccination HSV-2 serostatus.	2013	(54)
				Epstein-Barr Virus (EBV)	Phase II	Healthy young adults	gp350/AS04	The vaccine demonstrated a favorable safety profile and conferred 78% protective efficacy against EBV-induced infectious mononucleosis.	2007	(55)
	AS02	QS-21	Oil-in-water emulsion	Plasmodium falciparum	Phase IIb	Infants aged 8, 12, and 16 weeks	RTS,S/AS02D	Antibody levels against the circumsporozoite protein and hepatitis B surface antigen were consistently significantly higher in the RTS,S/AS02D group than in the control group. The vaccine conferred 50.7% and 26.7% protective efficacy against multiple malaria episodes at 12 and 18 months post-vaccination, respectively.	2013	(56)
				HIV	Phase II	HIV-infected individuals receiving stable, highly active antiretroviral therapy (HAART)	gp120/NefTat/AS02A	The vaccine demonstrated a favorable safety profile and induced robust gp120-specific CD4+ T cell responses.	2012	(57)
				HPV	Phase I/II	Healthy females aged 18–25 years	Quadrivalent HPV L1 VLP/AS04	The vaccine exhibited an acceptable safety profile. HPV-16/18 antibody titers induced by the quadrivalent AS04-adjuvanted vaccine were lower than those induced by Cervarix, a phenomenon associated with immune interference.	2014	(58)
	AS15	QS-21, CpG ODN	Liposomal formulation	Melanoma	Phase III	Patients with completely resected stage IIIB/IIIC MAGE-A3-positive cutaneous melanoma aged ≥18 years	MAGE-A3/AS15	The vaccine failed to improve disease-free survival or overall survival in patients with resected MAGE-A3-positive melanoma.	2018	(63)
				Non-small cell lung cancer (NSCLC)	Phase III	Patients with completely resected stage IB/II/IIIA MAGE-A3-positive NSCLC aged ≥18 years	MAGE-A3/AS15	The vaccine failed to improve disease-free survival in patients with resected MAGE-A3-positive NSCLC.	2016	(62)
				HER2-positive breast cancer	Phase I/II	Patients with HER2-overexpressing metastatic breast cancer	dHER2/AS15	The vaccine demonstrated a favorable safety profile. Among 40 patients, 2 achieved objective tumor responses and 10 had stable disease for ≥26 weeks.	2016	(91)
				Non-muscle-invasive bladder cancer	Phase I	Patients with non-muscle-invasive bladder cancer	MAGE-A3/AS15 + Bacillus Calmette-Guérin (BCG)	Bladder-localized T cell accumulation was significantly higher in the MAGE-A3/AS15 plus BCG group than in the MAGE-A3/AS15 alone group.	2017	(92)
MPL	ALFQ	QS-21	Liposomal formulation	Plasmodium falciparum	Phase I	Malaria-naive healthy adults	FMP013/ALFQ	The vaccine demonstrated a favorable safety profile and induced potent humoral and cellular immune responses superior to those elicited by the RTS,S/AS01 vaccine.	2022	(93)
				Severe Acute Respiratory Syndrome Coronavirus 2 (SARS-CoV-2)	Phase I	SARS-CoV-2 vaccine-naive adults	SpFN/ALFQ	The vaccine was well-tolerated and induced potent and durable binding and neutralizing antibodies against multiple SARS-CoV-2 variants.	2024	(94)
GLA	GLA-SE	None	Oil-in-water emulsion	Plasmodium falciparum	Phase I	Malaria-naive women aged 18–35 years and non-pregnant women naturally exposed to P. falciparum	VAR2CSA-DBL1x-2x/GLA-SE, VAR2CSA-DBL1x-2x/Alhydrogel	The vaccine exhibited an acceptable safety profile with no vaccine-related serious adverse events. It induced functional antibodies that bound homologous VAR2CSA, and antibody persistence was longer in the VAR2CSA-DBL1x-2x/GLA-SE group than in the VAR2CSA-DBL1x-2x/Alhydrogel group.	2020	(70)
				Mycobacterium tuberculosis	Phase IIa	Patients who had recently completed tuberculosis treatment	ID93/GLA-SE	Transcriptional changes in genes associated with innate immune signaling pathways were observed 3 days post-vaccination, while changes in genes related to lymphocyte expansion and B cell activation were detected 7 days post-vaccination.	2024	(71)
				Schistosoma mansoni	Phase I	Healthy male adults	Sm14/GLA-SE	The vaccine was well-tolerated with a favorable safety profile. It induced significantly elevated Sm14-specific IgG without IgE elevation and increased CD4+ T cells producing single cytokines (TNF-α, IL-2).	2016	(72)
				Mycobacterium leprae	Phase I	Healthy adults	LEP-F1/GLA-SE	The vaccine exhibited favorable tolerability and safety profiles and induced LEP-F1-specific antibodies and Th1 cytokines (IFN-γ, IL-2, TNF).	2020	(73)
				HIV	Phase I	Healthy infants ≤5 days of age born to HIV-infected mothers but HIV nucleic acid test-negative at birth	CH505TF gp120/GLA-SE	Local and systemic solicited adverse reactions were more frequent in the vaccine group than in the placebo group, and no vaccine-related serious adverse events were reported.	2025	(74)
				RSV	Phase II	Adults aged ≥60 years	RSV sF/GLA-SE, inactivated influenza vaccine (IIV)	Higher adjuvant doses increased injection site discomfort, but reactogenicity at the highest dose was similar to that of the inactivated influenza vaccine. Significant humoral and cellular immune responses were observed. The formulation containing 120 μg sF plus 5.0 μg GLA induced the highest responses in all participants and in elderly participants specifically.	2017	(75)
				Influenza virus	Phase I	Healthy adults aged 18–49 years	rHA/GLA-SE	After two doses, the proportions of subjects with hemagglutination inhibition titers ≥1:40 were 32% and 15% in the unadjuvanted 135 μg and 45 μg rHA groups, respectively, compared with 82%, 75%, 66%, and 72% in the groups receiving 45 μg, 15 μg, 7.5 μg, or 3.8 μg rHA plus GLA-SE, respectively. GLA-SE significantly increased serum-specific antibody titers.	2013	(76)
	GLA-AF	None	Aqueous formulation	Necator americanus	Phase I	School-age children	Na-GST-1/Alhydrogel + Na-APR-1/Alhydrogel/GLA-AF	The vaccine was well-tolerated and induced high levels of anti-Na-APR-1 and anti-Na-GST-1 IgG.	2024	(77)
				HIV	Phase II	Healthy individuals	CN54rgp140/GLA-AF + HIV-MVA	The CN54rgp140/GLA-AF plus HIV-MVA regimen induced significantly superior immune responses compared with HIV-MVA alone, with higher specific antibody titers and stronger cellular immune responses.	2018	(78)
				Schistosoma mansoni	Phase I	Non-endemic healthy adults	Sm-TSP-2/Alhydrogel/GLA-AF	The vaccine exhibited favorable tolerability and safety profiles. The Sm-TSP-2/Alhydrogel/GLA-AF group induced higher Sm-TSP-2-specific antibody levels and more durable immune responses than the Sm-TSP-2/Alhydrogel group.	2019	(79)
	GLA-LSQ	QS-21	Liposomal formulation	Plasmodium falciparum	Phase I	Healthy adults	rCSP/GLA-LSQ	All 26 participants who underwent controlled human malaria infection 28 days after the last vaccination developed malaria. Increasing vaccine doses induced higher immunoglobulin titers but did not reach the levels previously observed with RTS,S. The vaccine demonstrated insufficient protective efficacy.	2024	(80)

TLR4, Toll-like receptor 4; MPL, 3-O-deacylated monophosphoryl lipid A; QS-21, Quillaja saponaria fraction 21; HIV, Human Immunodeficiency Virus; HSV-2, Herpes Simplex Virus Type 2; EBV, Epstein-Barr Virus; RTS,S, recombinant *Plasmodium falciparum* circumsporozoite protein-hepatitis B surface antigen fusion protein; HAART, highly active antiretroviral therapy; HPV, Human Papillomavirus; VLP, virus-like particle; CpG ODN, CpG oligodeoxynucleotide; NSCLC, non-small cell lung cancer; BCG, Bacillus Calmette-Guérin; SARS-CoV-2, Severe Acute Respiratory Syndrome Coronavirus 2; TNF-α, tumor necrosis factor-alpha; IL-2, interleukin-2; IFN-γ, interferon-gamma; RSV, Respiratory Syncytial Virus; IIV, inactivated influenza vaccine.

The table summarizes the latest clinical progress of each adjuvant.

Comprehensive table showing the clinical development status of 12 TLR4 agonist adjuvants, including formulation types, target diseases, clinical trial phases and key results.

### 3.2 Bacterial enzymatic combinatorial chemistry

**Figure 3:**
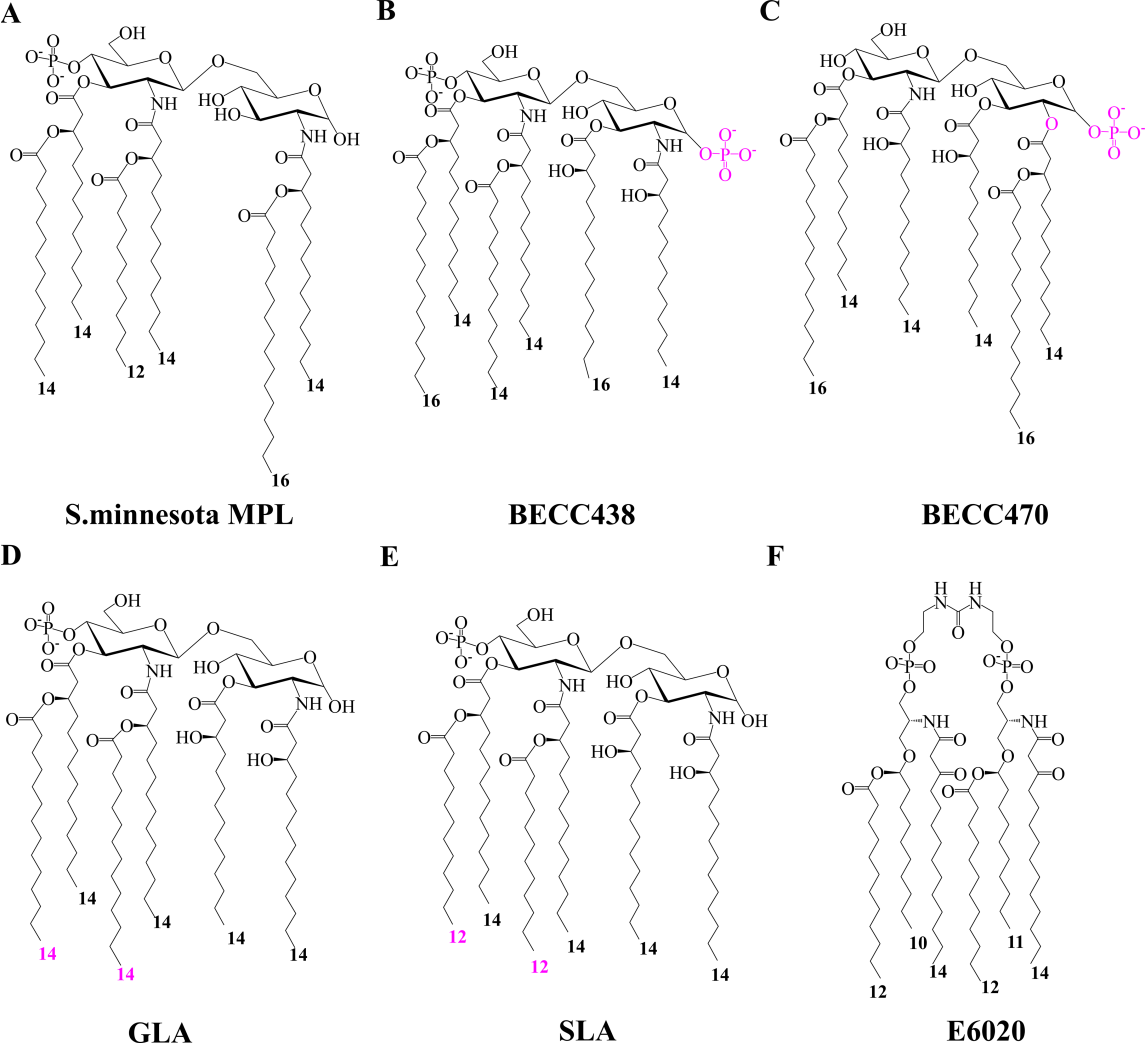
Chemical structures of *Salmonella minnesota*-derived MPL and TLR4 agonists BECC438, BECC470, GLA, SLA, and E6020. **(A)** *S. minnesota*-derived MPL. **(B, C)** BECC438 and BECC470: Bacterially engineered lipid A analog with two phosphate groups and mixed C14/C16 acyl chains. **(D)** GLA (glucopyranosyl lipid A): lipid A analog with six uniform C14 acyl chains (pink). **(E)** SLA (second-generation lipid A): optimized GLA derivative with two shortened C12 acyl chains (pink). **(F)** E6020: non-lipid A TLR4 agonist with a unique urea-linked dimer backbone and mixed C10–C14 acyl chains. Chemical structures of six TLR4 agonists: Salmonella-derived MPL, bacterially engineered BECC438 and BECC470, synthetic GLA with six C14 acyl chains, optimized SLA with two shortened C12 acyl chains, and non-lipid E6020 with a urea dimer backbone.

The Bacterial enzymatic combinatorial chemistry (BECC) platform was introduced in 2013 for the genetic engineering of bacteria to generate adjuvants with enhanced immunostimulatory potency and reduced toxicity. Specifically, structural modifications of the resulting LPS are achieved by deleting or introducing enzymes involved in LPS biosynthesis. These enzymes include acyltransferases, deacylases, phosphatases, and glycosyltransferases (64). Through this modular engineering strategy, a diverse panel of lipid A variants has been designed and generated, with BECC438 and BECC470 emerging as lead candidates with robust adjuvant activity and favorable safety profiles(**Figures 3B, C**) (64). Unlike MPL (derived from *Salmonella minnesota*), these adjuvants are recombinantly produced in *Yersinia pestis*. Structurally, BECC438 contains two phosphate groups and exhibits distinct acyl chain lengths relative to MPL (**Figure 3B**). Head-to-head comparisons of BECC438, BECC470, and the MPL analog GLA show that BECC compounds activate NF-κB signaling and induce cytokine production with significantly greater potency than GLA (64).

To evaluate the *in vivo* adjuvant activity of BECC438, mice received a two-dose regimen of *Yersinia pestis* rF1–V antigen formulated with BECC438, followed by bacterial challenge. Vaccinated mice were effectively protected against *Yersinia pestis* infection. Relative to GLA, BECC438 induced markedly higher rF1-V–specific total IgG levels after prime and boost immunizations (65). In an influenza vaccination study in aged mice, BECC470 combined with influenza hemagglutinin antigen conferred complete protection against viral challenge in 12-month-old animals. Furthermore, BECC470 elicited stronger immune responses than BECC438 or GLA (66). To date, however, neither BECC438 nor BECC470 has advanced to clinical trials.

### 3.3 *De novo*-synthesized MPL analogs

Although MPL is regulatorily approved and used in multiple vaccines, MPL derived from hydrolyzed LPS is a mixture with substantial batch-to-batch variability. This variability stems from two main factors: first, incomplete control of the hydrolysis process leads to inconsistent LPS hydrolysis; second, minor fluctuations in culture conditions cause subtle structural differences in bacterially produced LPS. Because MPL is produced via multi-step hydrolysis of bacterial biomass, contamination by residual bacterial components and LPS may occur. In contrast, *de novo* synthesis of TLR4 agonists does not require bacterial cultivation or downstream processing. Moreover, synthetic reactions can be precisely controlled, markedly lowering contamination risk and minimizing batch-to-batch variability. To date, several *de novo*–synthesized lipid A analogs have advanced to preclinical and clinical development.

#### 3.3.1 Glucopyranosyl lipid A

GLA, a *de novo*–synthesized lipid A analog, was developed by Avanti. It comprises a disaccharide backbone, one phosphate group, and six 14-carbon acyl chains. Structural differences between GLA and MPL lie mainly in acyl chain number, linkage positions, and length. GLA exhibits markedly stronger immunostimulatory potency than MPL (**Figure 3D**). In mouse models, GLA induces higher antibody titers than LPS or MPL and elicits potent type 1 T cell responses against HIV gag-p24 in the spleen and lymph nodes (67).

At present, GLA has been formulated into multiple adjuvant preparations: the oil-in-water emulsion GLA-SE, the aqueous formulation GLA-AF, and the QS-21-containing liposomal formulation GLA-LSQ. GLA-AF enhances immune responses induced by intradermal vaccination. Clinical studies have shown that intradermal inoculation facilitates the production of protective antibodies against Indonesian H5N1 (68, 69). Clinical studies have demonstrated that GLA-SE alone, without additional immunostimulants, confers protective immunity across malaria, tuberculosis, schistosomiasis,leprosy, HIV, RSV, influenza, and genital herpes (**Table 2**; 70–80). These findings demonstrate that a TLR4 agonist alone can generate protective immunity across diverse vaccine contexts.

#### 3.3.2 Second-generation lipid adjuvant

Driven by advances in protein structure determination and molecular docking, researchers have structurally optimized GLA based on ligand–receptor binding principles (81). Structural resolution and simulation of the GLA–TLR4 binding interface revealed that two of the six acyl chains in GLA are overly long. This feature allows GLA to bind the hydrophobic pocket of the TLR4–MD-2 complex but prevents optimal conformational fitting at the binding interface. Thus, although GLA activates TLR4 signaling, its agonist potency is not fully maximized. Subsequent work showed that removing one ethyl group from each of these two acyl chains at defined positions yields a molecule designated SLA, which has a larger hydrophobic surface area and higher binding affinity (**Figure 3E**) (82). SLA exhibits superior TLR4-binding and -activating capacity relative to GLA. In a murine herpes zoster vaccination study, the SLA oil-in-water emulsion (SLA-SE) was compared with Shingrix. In young mice, gE/SLA-SE induced gE-specific humoral and cellular immune responses comparable to Shingrix; in aged mice, gE/SLA-SE elicited stronger gE-specific cellular immune responses than Shingrix (83). Furthermore, SLA has shown appreciable efficacy in both infectious disease prevention and cancer therapy (84–86).

#### 3.3.3 E6020

Beyond lipid A–like TLR4 agonists, certain non-glycolipid compounds with no structural similarity to lipid A can also activate TLR4 and act as adjuvants. E6020 is a fully synthetic TLR4 agonist developed by Eisai Co., Ltd. Structurally, E6020 lacks a disaccharide backbone and contains two phosphate groups and six acyl chains (**Figure 3F**) (87). Relative to aluminum-containing adjuvants, an optimal dose of E6020 yields a 2-fold increase in peak antibody levels (88). In addition, E6020 can be combined with various adjuvants; for instance, co-formulation with MF59 emulsion significantly increases antibody titers. As a TLR4 agonist, E6020 boosts antibody titers and promotes antibody isotype switching after immunization with hapten-protein antigens (88). For cytomegalovirus vaccine development, researchers co-encapsulated QS-21 and E6020 into cholesterol-containing liposomes to create the Synthetic Adjuvant Platform 14 adjuvant. This adjuvant is well-tolerated and induces durable neutralizing antibody responses comparable to AS01B in both mice and non-human primates (89). In addition, E6020 has been used in vaccine development against pathogens including trypanosomes (89, 90).

## 4 Perspectives

The clinical value of TLR4 agonists as vaccine adjuvants has been firmly established by the successful translation of MPL—the first clinically approved TLR4 agonist—into licensed vaccines against herpes zoster and respiratory syncytial virus. Global research efforts have yielded a diverse portfolio of TLR4 agonist development strategies, including natural lipid A structural modification, bacterial genome engineering, structure-based *de novo* synthesis, and small-molecule agonist discovery via high-throughput screening and computer-aided drug design. Concurrently, advances in formulation technology have accelerated the clinical translation of TLR4 agonists. Formulation platforms including liposomes, oil-in-water emulsions, and nanosuspensions, have mitigated key inherent limitations of TLR4 agonists—poor aqueous solubility, limited bioavailability, and elevated reactogenicity—by enhancing *in vivo* delivery efficiency and stability, significantly expanding their translational potential. Accumulating clinical trial data demonstrate that most next-generation TLR4 agonists exhibit improved immunostimulatory potency and favorable safety profiles, supporting their utility in both infectious disease prevention and cancer immunotherapy.

Nevertheless, several challenges remain in translating next-generation TLR4 agonist candidates to the clinic. First, the therapeutic window between immunostimulation and systemic reactogenicity remains narrow. Although MyD88-driven pro-inflammatory cytokine production is a major contributor to adverse reactions such as fever and influenza-like symptoms—as exemplified by the higher reactogenicity rates of AS01 relative to aluminum-based adjuvants—reactogenicity is also modulated by TRIF signaling, formulation components, and host factors. Current strategies, including TRIF-biased agonist design and formulation approaches that confine TLR4 agonist activity to the injection site, have shown preclinical promise, but robust clinical evidence that these approaches can meaningfully reduce reactogenicity without compromising adjuvant potency is still lacking for most candidates. Second, the compatibility of TLR4 agonists with non-protein vaccine platforms remains poorly characterized. Most clinical experience derives from recombinant protein-based vaccines, yet mRNA, DNA, and viral vector platforms possess distinct innate activation profiles and may not benefit from TLR4 co-stimulation in the same manner. Systematic studies addressing whether and how TLR4 agonism can enhance the immunogenicity of these increasingly dominant platforms are urgently needed. Third, manufacturing challenges persist. MPL is a heterogeneous mixture with inherent batch-to-batch variability, while *de novo*-synthesized agonists such as GLA and SLA offer greater homogeneity but involve complex multi-step synthesis that currently limits scalability. Bridging the gap between chemical precision and industrial feasibility remains a priority.

Addressing these challenges through continued advances in agonist design, formulation optimization, and manufacturing technology will expand the clinical utility of TLR4-targeted adjuvants across the evolving vaccine landscape.
